# Polyol–mediated synthesis of nickel oxide nanoparticles through aqueous sol–gel route

**DOI:** 10.1186/s13065-022-00898-8

**Published:** 2022-11-27

**Authors:** Samreen Zahra, Waneeza Shahid, Chaudhry Athar Amin, Sarwat Zahra, Bushra Kanwal

**Affiliations:** 1 Mineral Processing Research Centre, Pakistan Council of Scientific and Industrial Research Laboratories Complex, Ferozepur Road, Lahore, 54600 Pakistan; 2 Department of Chemistry, Post Graduate Islamia College, Cooper Road, Lahore, 54000 Pakistan; 3 Department of Physics, University of Education, College Road Township, Lahore, 54770 Pakistan; 4 Centre for High Energy Physics, University of the Punjab, Lahore, 54590 Pakistan

**Keywords:** Nickel oxide, Nanoparticles, Polyol, Sol–gel process, Glycerol, Calcination

## Abstract

**Background:**

In this work, nickel oxide nanoparticles were prepared by polyol mediated aqueous route of sol–gel process using nickel nitrate hexahydrate as precursor, a mixture of isopropyl alcohol and water as solvent and glycerol for making polyol medium followed by calcination at various temperatures ranging from 500 to 900 °C. Characterization was carried out using X–ray diffractometry, infrared spectroscopy, differential scanning calorimetry-thermogravimetry and field emission scanning electron microscopy.

**Results:**

The results confirmed the formation of face-cantered cubic structure of nickel oxide with its complete conversion after calcination at 900 °C; significant variation in the surface morphology was observed with the increasing calcination temperature.

**Conclusions:**

The study revealed that the aqueous sol–gel route using polyol system followed by calcination at ambient temperatures lead to the successful synthesis of nickel oxide nanoparticles.

## Introduction

Nickel oxide nanoparticles are one of the most promising materials for technological applications such as magnetic materials, ion storage materials, battery electrodes, photo–electron devices, thermoelectric materials, fuel cells, gas sensors, catalysts, dye–sensitized photocathodes, electrochromic films and non–enzymatic glucose sensors etc [1]. Nickel oxide is among those metal oxides that exhibit p–type nature and possesses a wide band gap ranging from 3.6 to 4.0 eV due to which it can be employed as a transparent p–type semiconducting layer [2, 3]. In particular, nano–dimensional particles because of their large surface area, display a wide range of interesting size–dependent electro–optical, magneto–optical, chemical and mechanical properties [1]. Research attention has therefore been focused by the scientists on different routes for the synthesis of nickel oxide nanoparticles. Generally, nanoparticles can be prepared by physical, chemical and biological methods. The chemical and physical methods include co–precipitation method, sol–gel technique, microemulsion method, solvothermal synthesis, ultrasonic radiation, microwave irradiation and anodic arc plasma method. In addition, biological methods comprising the use of biological materials such as different parts of plants, fungi, algae, bacteria, and actinomycetes species have also been employed [4, 5].

Synthesis of nanostructures with controlled morphology including prevention of agglomeration and oxidation of nanoparticles keeping in view their bulk production is of extensive research interest these days. Sol–gel method is the easiest one for tuning the morphology of nanoparticles simply by regulating the process parameters [6]. Another approach for adjusting the size and shape of nanostructures is by using some organic solvent or a surfactant capable of inhibiting agglomeration and thus producing well–defined monodispersed structures [7]. According to Rakshit et al*.* morphology controlled process for the transformation of precursors is the most favorable method for the preparation of nickel oxide nanostructures [8]. Niasari et al*.* reported the synthesis of nanoparticles of NiO using nickel oxalate and oleylammine as precursors [9]. Synthesis of nanosize NiO by heat treatment of nickel octanoate has also been reported by Niasari et al. [10]. Stabilizers like polyvinylpyrrolidone (PVP) surfactant are often added for particle protection and controlled﻿ particle size [11]. Zhang et al*.* prepared nickel oxide fibres through PVP approach [12]. Xu et al*.* synthesized NiO nanorods by using thermal decomposition method followed by a surfactant nonyl phenyl ether (NP-9/5) base process and NaCl flux at 1173 K [13]. Hence, it is important and significant to synthesize nickel oxide nanoparticles with a facile pathway. During last few decades, nano size nickel oxide particles have been successfully synthesized using sol–gel method which is believed to be the simplest and the most economical technique. Teoh et al*.* synthesized nickel oxide nanoparticles by surfactant mediated approach of sol–gel process using poly (alkylene oxide) copolymer [14].

This work describes the synthesis of nickel oxide nanoparticles by aqueous sol–gel route using glycerol as polyol for a better control on the morphology of nanoparticles, followed by calcination at ambient temperatures. The effect of temperature variation on crystal structure, surface functional groups, thermal behavior and surface morphology of the synthesized nanoparticles is determined by using XRD, IR, DSC–TGA and FESEM.

## Experimental

### Reagents

Nickel nitrate hexahydrate (Thermo Scientific 99%), isopropyl alcohol (WINLAB 99.9%), glycerol (99%) and nitric acid (Merck 70%) were used.

### Synthesis of nickel oxide nanoparticles

**Fig. 1:**
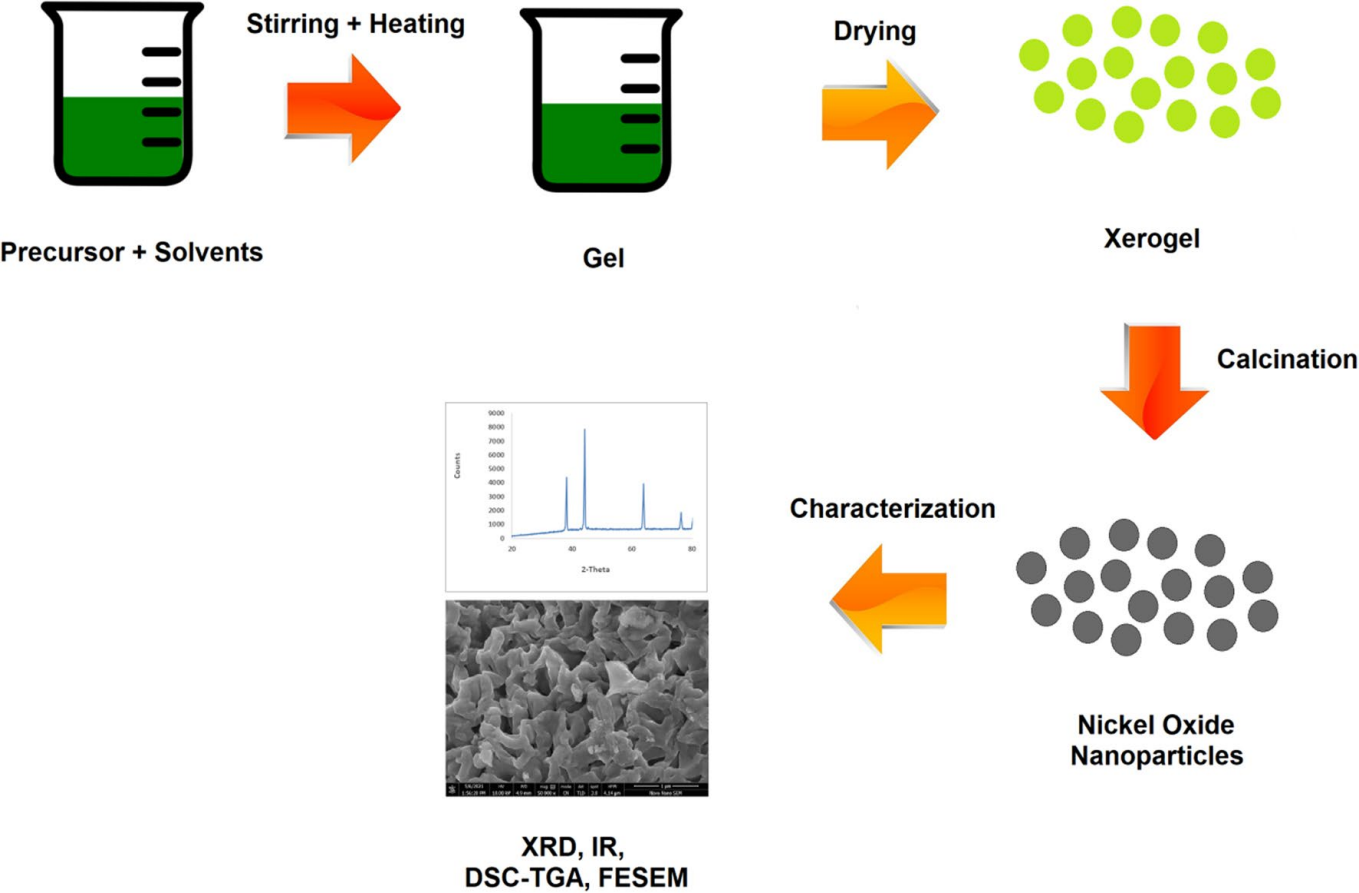
Graphical representation of sol–gel process

Nickel oxide nanoparticles were synthesized by aqueous sol–gel route in acidic medium. 19.45 g nickel nitrate hexahydrate precursor was added to a mixture of solvents i.e. isopropyl alcohol and water in 1:8 ratio. 10 mL of glycerol were added and pH of the solution was maintained at 1 using nitric acid. The mixture was stirred constantly for 2 h at 70 °C. The sol thus formed was dried at 80 °C for several hours in order to dry it completely to obtain gel which was ground well. The powdered sample obtained﻿ was divided into five portions that were calcined for 2 h at 500, 600, 700, 800 and 900 °C and were labeled as NN–1, NN–2, NN–3, NN–4 and NN–5 respectively. The graphical representation of the process is shown in Fig. 1.

### Characterization

The crystalline nature of nickel oxide nanoparticles was investigated by X–ray diffraction technique. The XRD patterns of the prepared samples were recorded with the help of Bruker D2 PHASER X–ray diffractometer using monochromatised CuK_α_1 radiation at a wavelength of 1.54060 *Å*. IR spectra were recorded with Thermo Nicolet IR 200 (USA). Thermal behavior was studied using differential scanning calorimeter Universal V4.5A, TA instruments USA, varying the temperature from room temperature to 1000 °C at a rate of 10 °C/min in nitrogen atmosphere. Structural studies of the samples were done by FESEM FEI Nova 450 NanoSEM.

## Results and discussion

Synthesis of nickel oxide nanoparticles was carried out using glycerol for making polyol medium because of its lower cost and nontoxicity. The polyol medium significantly controls the formation of hard agglomerates during the synthesis of metal oxide nanoparticles obtained through aqueous sol–gel routes; therefore the use of high boiling polyols is more appropriate. Glycerol has also been reported to be used for the reduction of metal salt precursors for the preparation of various metal nanoparticles like silver, tantalum, palladium, platinum, ruthenium and gold. Moreover, manganese hydroxide, manganese carbonate and vanadium oxide have been synthesized in the presence of glycerol as a reducing agent [15]. Hence, glycerol added to a mixture of isopropanol and water acts as a solvent as well as a mild reducing agent leading to slow transformation of nickel alkoxide intermediate to nickel oxide nanoparticles.

### X-ray diffraction analysis

**Fig. 2:**
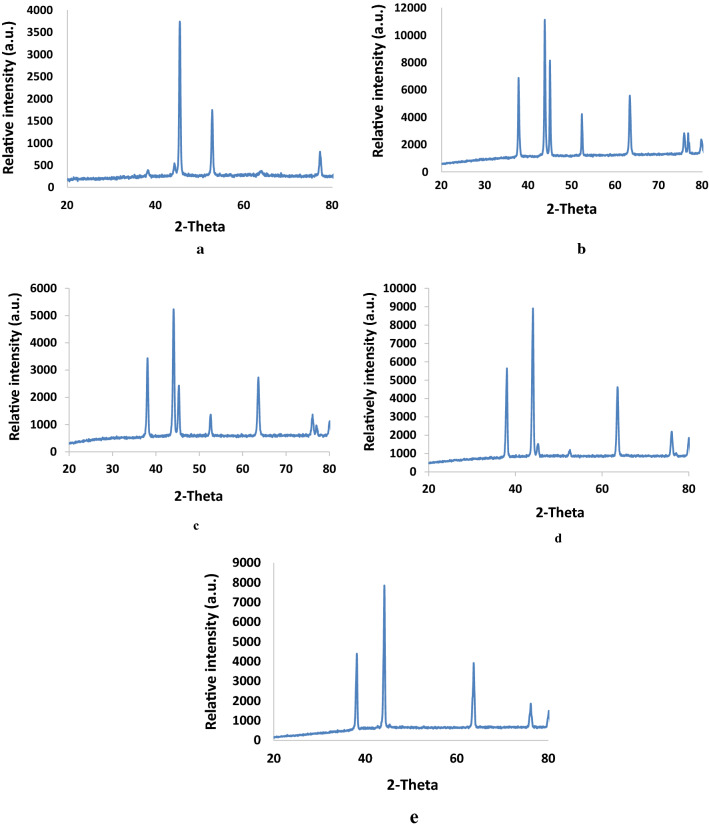
**a** XRD pattern of NN–1. **b** XRD pattern of NN–2. **c** XRD pattern of NN–3. **d** XRD pattern of NN–4. **e** XRD pattern of NN–5

The phase composition of synthesized nanoparticles NN–1, NN–2, NN–3, NN–4 and NN–5 was studied using X–ray diffraction analysis and the diffraction patterns are presented in Fig. 2a–e. The diffraction peaks for NN–1 at 2θ degrees of 45.37, 52.76 and 77.08° correspond to the (111), (200), and (220) crystal faces of Ni as compared with the standard JCPDS data reported in JCPDS card No. 04–0850 [16]. The sharp peaks showed the formation of nickel alkoxide intermediate at 500 °C. The pattern for NN–2 exhibited additional distinct peaks at 37.68, 43.70, 63.22, 75.72 and 79.61° relative to (111), (200), (220), (311), and (222) crystal planes of NiO respectively compared with the reported standard JCPDS data for NiO (JCPDS card No: 78–0429) which revealed partial conversion of the intermediate into nickel oxide at 600 °C [17, 18]. NN–3 and NN–4 displayed diffraction patterns similar to NN–2 with slight variation in intensities of peaks indicating the gradual increase in conversion of intermediate to nickel oxide at 700 and 800 °C leading to its complete transformation to well–crystallized single phase face centered cubic geometry with lattice constants, a = b = c = 4·1771* Å* at 900 °C.

### Infrared spectroscopy

**Fig. 3:**
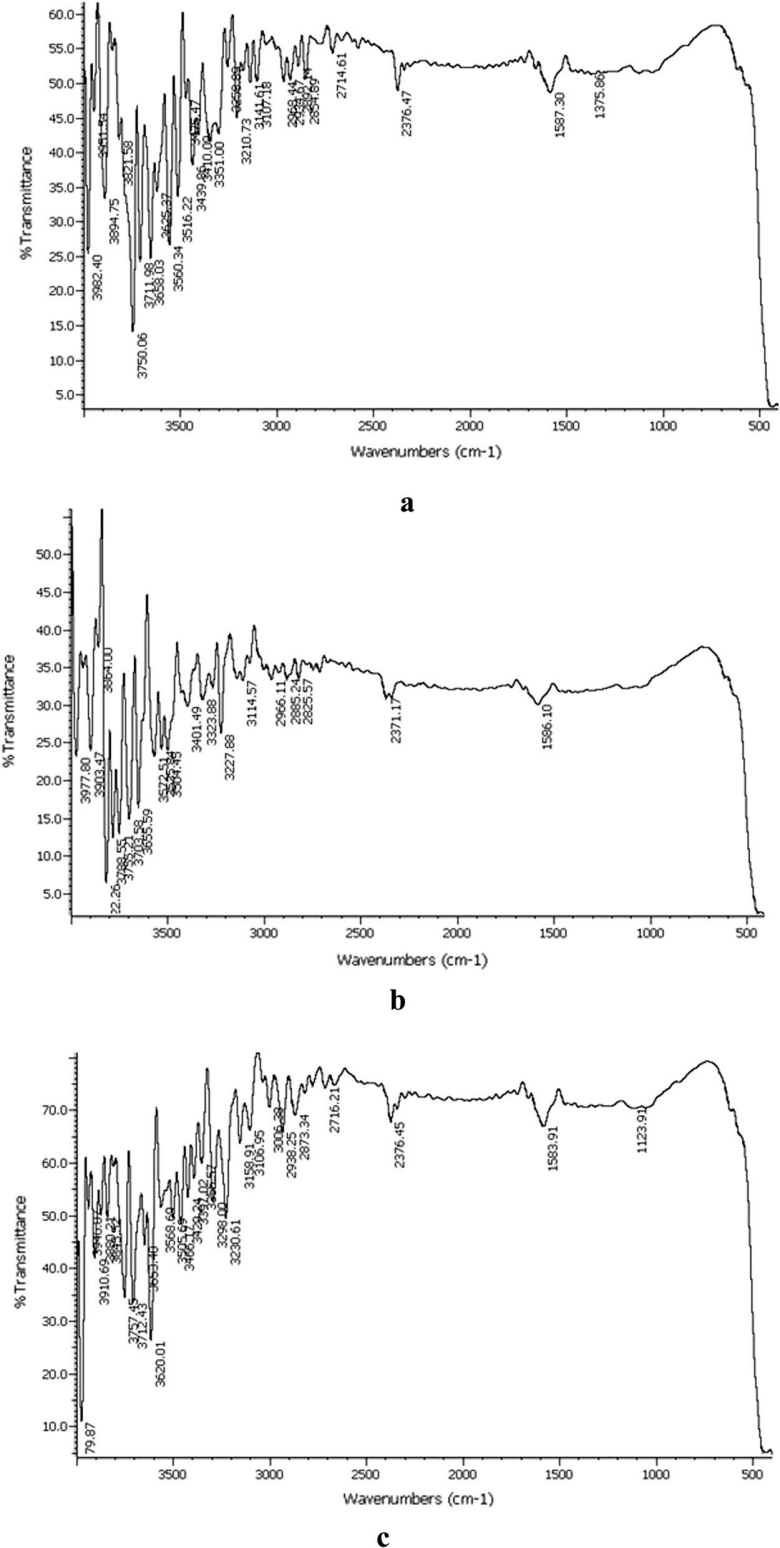

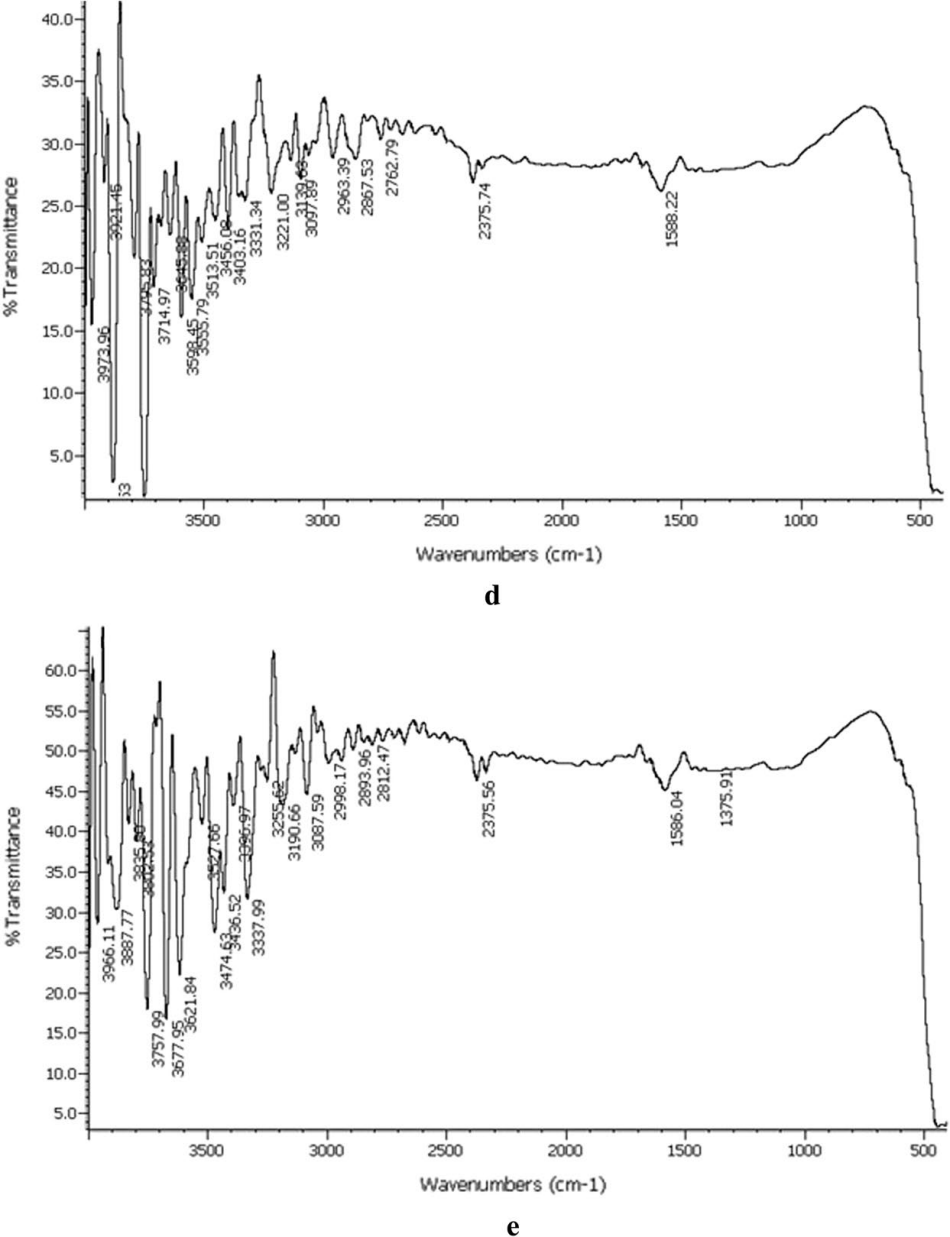
**a** IR spectrum of NN–1. **b** IR spectrum of NN–2. **c** IR spectrum of NN–3. **d** IR spectrum of NN–4. **e** IR spectrum of NN–5

Figure. 3a–e exhibit the infrared spectra of NN–1, NN–2, NN–3, NN–4 and NN–5 recorded in the range of 4000–400 cm^−1^. The figures illustrate similar spectra for all the prepared samples comprising various significant absorption bands. The sharp absorption bands in the region between 3800 and 3000 cm^−1^ are attributed to the O–H stretching vibrations of hydroxyl groups. The appearance of such bands represents the adsorption of water molecules on the external surface of nanoparticles in addition to hydrogen bonded hydroxyl groups. The weak bands between 3000 and 2800 cm^−1^ correspond to the stretching vibrations of alkyl groups of organic impurities entangled by the nanocrystals during their formation [19]. Another weak band at 1587.30 cm^−1^ is assigned to H–O–H bending vibrational modes [20].

The prominent absorption band in the region below 1000 cm^−1^ is ascribed to the stretching modes of metal–oxygen bonds that substantiate the presence of Ni–O. The broadness of this band indicates the nanosize of nickel oxide nanoparticles [21]. Similar strong absorption bands below 1000 cm^−1^ have been reported for nickel oxide nanoparticles by the scientists in the past [22–24].

### Thermal analysis

**Fig. 4:**
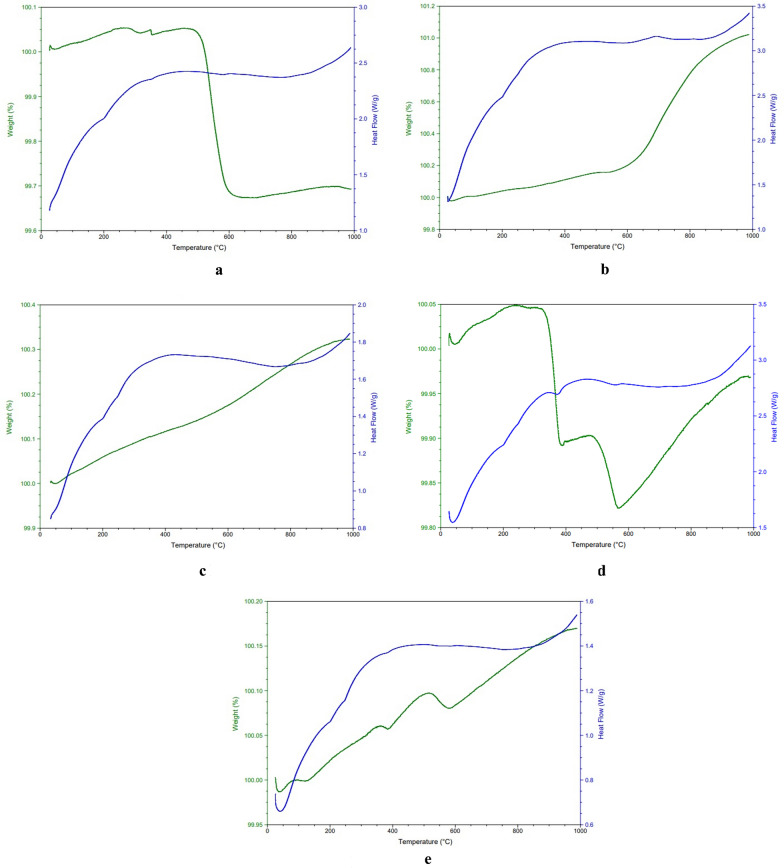
**a** DSC–TGA curves of NN–1. **b** DSC–TGA curves of NN–2. **c** DSC–TGA curves of NN–3. **d** DSC–TGA curves of NN–4. **e** DSC–TGA curves of NN–5

Thermal behavior of synthesized NN samples was studied by DSC–TGA and curves are illustrated in Fig. 4a–e. The curves exhibit negligible weight gain and weight loss ranging from 0.1 to 0.34% from room temperature to 1000 °C. The heat flow curves depict all the weight changes accompanied with their corresponding endothermic and exothermic peaks shown by the NN samples. These heat changes can be attributed to the removal of adsorbed moisture, organic residues entrapped between the crystals during their synthesis as well as the heat transfer between sample and crucible. According to previous researchers the endothermic reactions are attributed to the decomposition of water, whereas the exothermic reactions occur due to the oxidation of the decomposed product [25]. Most of the studies have reported negligible weight loss for nickel oxide nanoparticles above 400 °C indicating the formation of thermally stable product [26].

### Field emission scanning electron microscopy

**Fig. 5:**
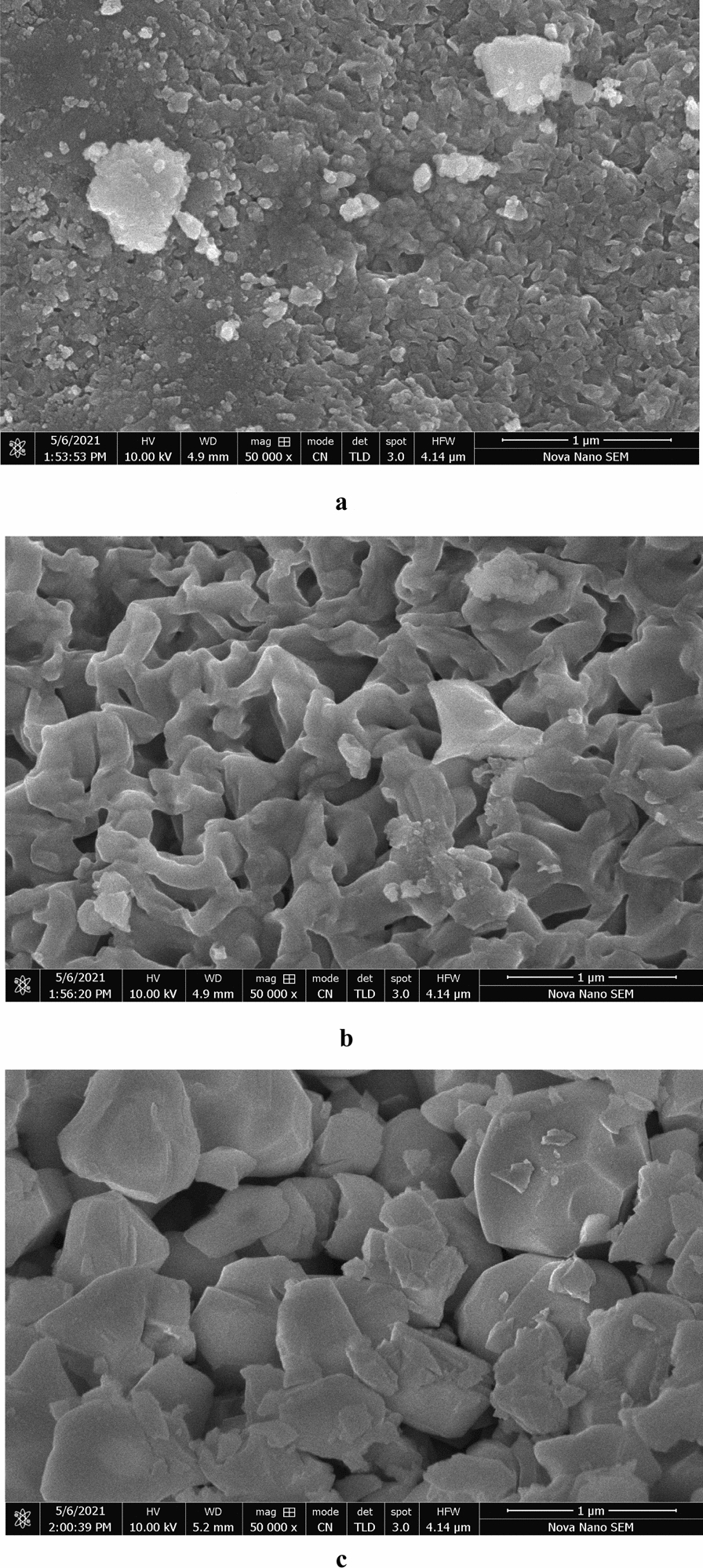
**a** FESEM micrograph of NN–1. **b** FESEM micrograph of NN–3. **c** FESEM micrograph of NN–5

The surface morphology of nickel oxide nanoparticles was investigated using field emission scanning electron microscopy and the images recorded at 50,000 × magnification are presented in Fig. 5a–c. The as–prepared nanoparticles show aggregated structures of irregular shape having size in the range of several nanometers; the aggregation rate of the nanoparticles is believed to be the structure and morphology determining factor for the nanoparticles. The results show that NN–1 demonstrates higher aggregation that decreases with the increasing calcination temperature in case of NN–3 and NN–5 due to intensified nucleation rate of nanoparticles. The rate of nucleation is greatly influenced by high temperature due to accelerated core formation after attaining super–saturation of the product. The particle size on the other hand, increases due to quick particle growth at higher calcination temperatures resulting in the formation of flakes and hence, NN–5 bares flake–like morphology with reduced aggregation.

Figure. 5b clearly depicts that NN–3 exhibits quite different morphology with elongated structures combined to form a network due to aggregation of particles. According to Lim et al*.* cylindrical rod–like structures of nickel oxide go through a two–step process, a first step comprising nucleation of atoms formed by metal salt reduction and another step of crystal growth from nuclei through atomic growth [27]. The other reason for aggregation of nickel oxide nanocrystals often noticed by the former scientists is their high surface area and high surface energy attained during sol–gel synthesis [14]. Moreover, nanosize nickel oxide has greater tendency to aggregate due to the fact that it is antiferromagnetic in nature; this aggregation resulting in increased diameter leads to enhanced magnetic moment of nickel oxide nanoparticles [17]. Bani–Fawaz et al*.* obtained a three–dimensional network of randomly oriented structures of nickel oxide aggregated without significant spacing between individual nanoparticles [28]. Uddin et al*.* also confirmed highly agglomerated structures of synthesized nickel oxide nanoparticles [29]. Further comparison of morphology of nickel oxide nanoparticles prepared by different routes is given in Table 1.

**Table 1:** Comparison of morphology of nickel oxide nanoparticles prepared by different routes

Synthesis method	Shape	Size	References
Wet chemical	Spherical/agglomerated	5–120 nm	Hong et al*.* [1]
Sol–gel	Cubic	74.5 nm	Ghazal et al*.* [21]
Microwave combustion	Spherical/agglomerated	19–30 nm	Anand et al*.* [30]
Hot plate combustion	Cylindrical and rod-like/self-aggregated	25–30 nm	Ezhilarasi et al*.* [31]
Chemical co–precipitation	Spherical/agglomerated	18.3 nm	Sagadevan et al*.* [32]
Chemical capping	Spherical/agglomerated	97 nm	Rifaya et al*.* [33]

## Conclusion

Nickel oxide nanoparticles were successfully synthesized by aqueous sol–gel route adopting glycerol for making polyol system. The method employed for the synthesis of nanoparticles is quite simple, economical and operates under mild conditions making the process suitable for large scale production. Characterization of the as–prepared nickel oxide nanoparticles revealed the formation of face–cantered cubic structure of nickel oxide with its complete conversion after calcination at 900 °C. However, high aggregation of particles was observed that decreased with the rise in calcination temperature resulting in the formation of flakes of irregular shape and different sizes in several nanometers range.

## Data Availability

The datasets generated and/or analyzed during the current study are not publicly available in order to avoid their misuse but are available from the corresponding author on reasonable request.
